# Cannabidiol in the Treatment of Epilepsy: A Focused Review of Evidence and Gaps

**DOI:** 10.3389/fneur.2020.531939

**Published:** 2020-10-19

**Authors:** Guilherme Diogo Silva, Felipe Borelli Del Guerra, Maira de Oliveira Lelis, Lécio Figueira Pinto

**Affiliations:** ^1^Department of Neurology, Hospital das Clínicas da Faculdade de Medicina da Universidade de São Paulo, São Paulo, Brazil; ^2^Federal University of Amazonas, Manaus, Brazil

**Keywords:** cannabidiol, epilepsy, safety, efficacy, gaps, evidence, review

## Abstract

Approximately one third of epilepsy patients do not become seizure free with antiseizure medications. This treatment gap motivates research for new therapeutic options, such as cannabidiol (CBD). CBD differs from other cannabis derivatives because of its consistent efficacy and lack of a psychoactive effect. CBD can be recommended as adjunctive therapy in patients with Dravet and Lennox-Gastaut syndromes. The most common adverse effects (AEs) are drowsiness, reduced appetite, diarrhea, and vomiting. Transaminase elevation is the most common AE that leads to CBD discontinuation. Coadministration with valproate may increase the risk of hepatotoxicity. The combination of CBD and clobazam may increase both the effectiveness and the risk of AEs associated with these drugs. The most striking gaps in knowledge are the efficacy and optimal dose of CBD for adults with focal epilepsies, the long-term safety of CBD use, and strategies to improve access to CBD for people living with epilepsy.

## Introduction

Epilepsy can be a therapeutic challenge. Despite the growing number of antiseizure medications (ASMs), approximately one third of patients with epilepsy have persistent seizures (1). Surgical treatment, although still underused, may be an alternative in up to 25% of these cases (2). Therefore, many patients are not seizure free. This treatment gap motivates research on new ASMs, such as cannabidiol (CBD).

The medical use of marijuana has gained considerable interest in the press in the last two decades. Three reasons for this are (a) the appeal of being a “natural” alternative treatment (3); (b) the discovery of a complex cell-signaling system responsive to cannabis, the endocannabinoid system (4); and (c) prominent public cases, such as Charlotte Figi in the United States (5).

Cochrane and American Academy of Neurology reviews determined that there was no scientific evidence to support the use of cannabis for epilepsy in 2014 (6, 7). At that time, there were only four placebo-controlled studies on cannabinoid use in epilepsy (8–11). All studies show inadequate power and methodological problems, but despite this, there has been an increasing use of CBD for the treatment of epilepsy (12).

Cannabinoids are obtained from different species of cannabis. Tetrahydrocannabinol (THC) and cannabidiol (CBD) are two of the most prominent cannabinoids found in the Cannabis plant (3). THC is responsible for the psychoactive effects of marijuana, but studies on its effects on epilepsy have shown conflicting results (13–16). THC binds to type 1 cannabinoid receptors (CB1) present in the basal ganglia, cerebellum, hippocampus, hypothalamus, and limbic system. Anandamide and 2-arachidonoylglycerol are endogenous cannabinoid agents that act on presynaptic CB1 and cause a reduction in excitatory activity. As THC is a partial agonist, this could explain the proconvulsant effect.

CBD is a cannabinoid that lacks psychoactive effects. It has a more consistent antiepileptic efficacy than THC (17, 18). CBD does not activate cannabinoid receptors. It does, however, interact with several other signaling systems. Transient receptor potential vanilloid type 1 (TRPV1)-mediated signaling may be the most relevant pathway in the anticonvulsant effect of CBD (19–21).

## Evidence on Effectiveness

In 2016, Dr. Orrin Devinsky presented an open-label study with 214 pharmacoresistant child-onset epilepsy patients who received CBD. Dravet (20%) and Lennox-Gastaut (19%) syndromes were the most frequent causes. The initial dose of 5 mg/kg/day was increased up to a maximum dose of 50 mg/kg/day if tolerated. The median monthly frequency of motor seizures was 30.0 at baseline and was reduced to 15.8 over the 12-week treatment period (22).

Another open-label study was performed in patients with pharmacoresistant epilepsy with tuberous sclerosis who received CBD. The initial dose of 5 mg/kg/day was increased by 5 mg/kg/day every week up to a maximum dose of 50 mg/kg/day if tolerated. The median reduction in total weekly seizure frequency was 48.8% after 3 months of treatment (23).

In 2017, the first randomized, double-blinded, placebo-controlled study evaluating high-purity CBD in patients with Dravet syndrome was published (24). The intervention group received a highly pure 100 mg/ml CBD solution. The dose was increased up to 20 mg/kg. The percentage of patients who had at least a 50% reduction in convulsive-seizure frequency was 43% after a 14-week treatment period with CBD. The overall conditions improved by at least one category on the Caregiver Global Impression of Change scale for 62% of patients in the CBD group. Three patients in the CBD group were seizure free. There was no significant reduction in nonconvulsive seizures.

In 2018, a randomized, double-blind, placebo-controlled study was published on patients with Lennox-Gastaut syndrome who used CBD (25). The patients had two or more drop seizures per week and a mean age of 16 years. Favorable outcomes were found in the 10 and 20 mg/kg CBD groups during the treatment period with a median percentage reduction from baseline in the frequency of drop seizures of 37.2 and 41.9% in the 10 and 20 mg/kg CBD groups, respectively.

Secondary outcomes were also significant. Thirty-six percent and 39% of patients had at least a 50% reduction from their baseline in drop-seizure frequency in the CBD groups compared with 14% in the placebo group. Furthermore, compared with the placebo group, a greater percentage of patients had at least a 75% reduction from baseline in drop-seizure frequency (11 and 25% in the CBD groups, 3% with placebo). Some patients became free from drop seizures during the entire maintenance phase in the CBD groups (4% and 7%, 1% in the placebo group).

The estimated median difference in reduction from baseline in the frequency of all seizures was 19.5 (*p* = 0.002) and 18.8 (*p* = 0.009) percentage points in the 10 and 20 mg CBD groups, respectively. Additionally, an improvement from baseline in overall condition according to the Patient or Caregiver Global Impression of Change at the last visit was reported in 66 and 57% of 10 and 20 mg CBD-treated patients, respectively, compared to 44% in the placebo group with an odds ratio of 2.57 (*p* = 0.002) for the 10-mg cannabidiol group vs. the placebo group and 1.83 (*p* = 0.04) for the 20 mg cannabidiol group.

Another randomized, double-blind, placebo-controlled phase 3 trial investigated the efficacy of CBD as an add-on therapy for drop seizures in patients with treatment-resistant Lennox-Gastaut syndrome. The results confirmed the efficacy of CBD with a median percentage reduction in monthly drop seizure frequency from a baseline of 43.9% in patients treated with 20 mg/kg of CBD compared to 21.8% in the placebo group. Other secondary outcomes were positive, including a greater proportion of patients experiencing a reduction of ≥75% seizures during the treatment period (20% CBD vs. 8% on placebo; *p* = 0.0273) (26).

A systematic review identified six randomized controlled studies (27). The average age of the participants was 16.1 years (0.5–55 years). At a dose of 20 mg/kg/day, the number needed to treat to one person experiencing 50%+ seizure reduction was 8. CBD was more effective than placebo at achieving complete seizure freedom. In 14 observational studies, 8.5% of patients were seizure free (95% CI, 3.8–14.5%).

Another recent systematic review (28) evaluated the role of concomitant clobazam (CLB) use on the efficacy of CBD in patients with Dravet syndrome and Lennox-Gastaut syndrome and enrolled 714 participants in four trials (429 treated with CBD, 240 with taking concomitant CLB). The percentages of patients not taking CLB who had at least a 50% reduction in seizure frequency during the treatment period were 29.1% with CBD and 15.7% in the placebo group (RR 1.80, *p* = 0.015); among patients receiving CLB, a 50% reduction in seizure frequency was achieved by 52.9 and 27.8% in the CBD and placebo groups, respectively (RR 1.85, *p* < 0.001). This study suggests that despite the drug–drug interactions that occur between CBD and CLB, adjunctive treatment with CBD can reduce seizures independent of concomitant CLB, reinforcing that CBD has intrinsic antiseizure activity (28).

Available data indicate that patients cotreated with CBD and CLB have higher response rates, highlighting that both pharmacodynamic and pharmacokinetic interactions may contribute to the efficacy of this combination (29).

**Figure 1:**
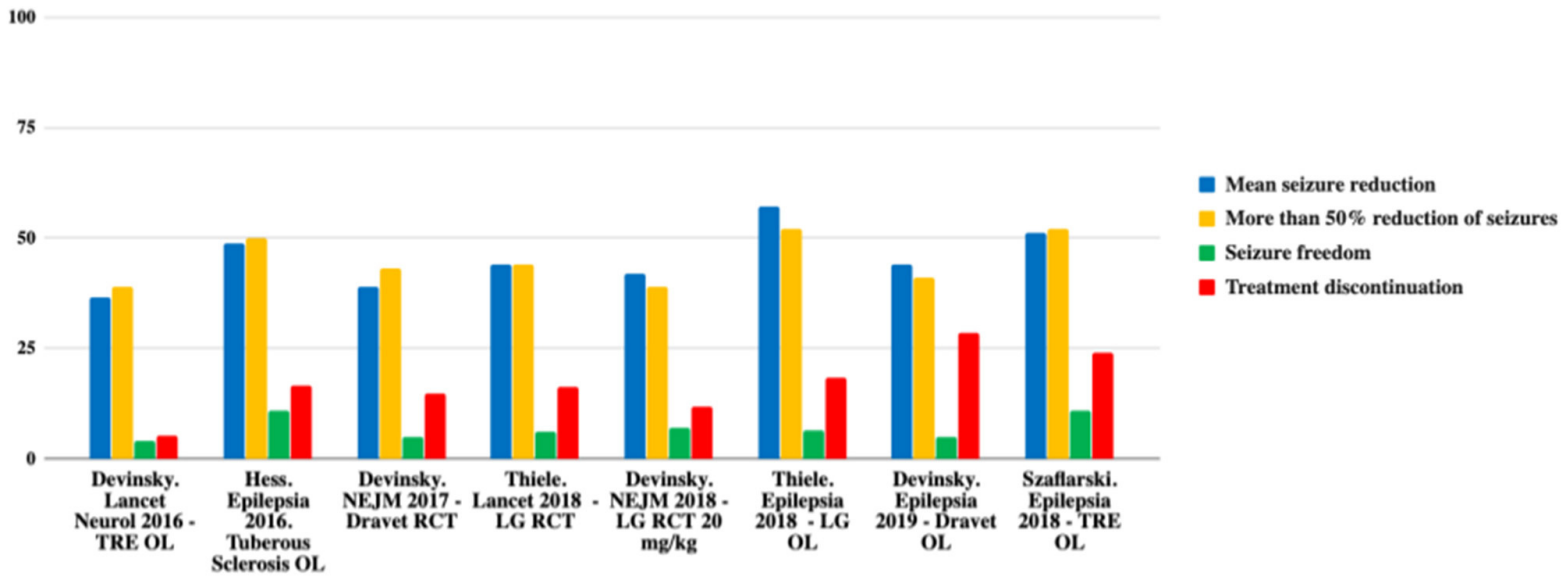
Comparison of efficacy in the main studies. TRE, treatment-resistant epilepsy; OL, open label; LG, Lennox-Gastaut; RCT, randomized controlled trial. Adapted from (12).

Therefore, CBD is effective as an adjunctive therapy in the treatment of drug-resistant childhood-onset epilepsy. Nevertheless, current evidence is restricted to rare and severe epileptic syndromes. A summary of CBD effectiveness can be seen in Table 1 and Figure 1 (12).

**Table 1:** Major studies about CBD in the treatment of epilepsy.

**Study**	**Number of patients**
Devinsky et al. (22) - TRE OL	214
Hess et al. (23) - Tuberous sclerosis OL	18
Devinsky et al. (24) - Dravet RCT	61
Thiele et al. (26) - LG RCT	86
Devinsky et al. (25) - LG RCT 20 mg/kg	76
Thiele et al. (30) - LG OL	366
Devinsky et al. (31) - Dravet OL	264
Szaflarski et al. (32) - TRE OL	607

*TRE, treatment-resistant epilepsy; OL, open label; LG, Lennox-Gastaut; RCT, randomized controlled trial*.

## Safety

We do not recommend cannabis or other THC-rich compounds because their effect on seizure control is uncertain, and they have psychotropic effects (12). They may present a risk of dependence, depression, psychosis, and suicide attempt. Indeed, it is estimated that up to 50% of first-episode psychosis could be prevented by controlling cannabis exposure in some countries (20, 33).

**Figure 2:**
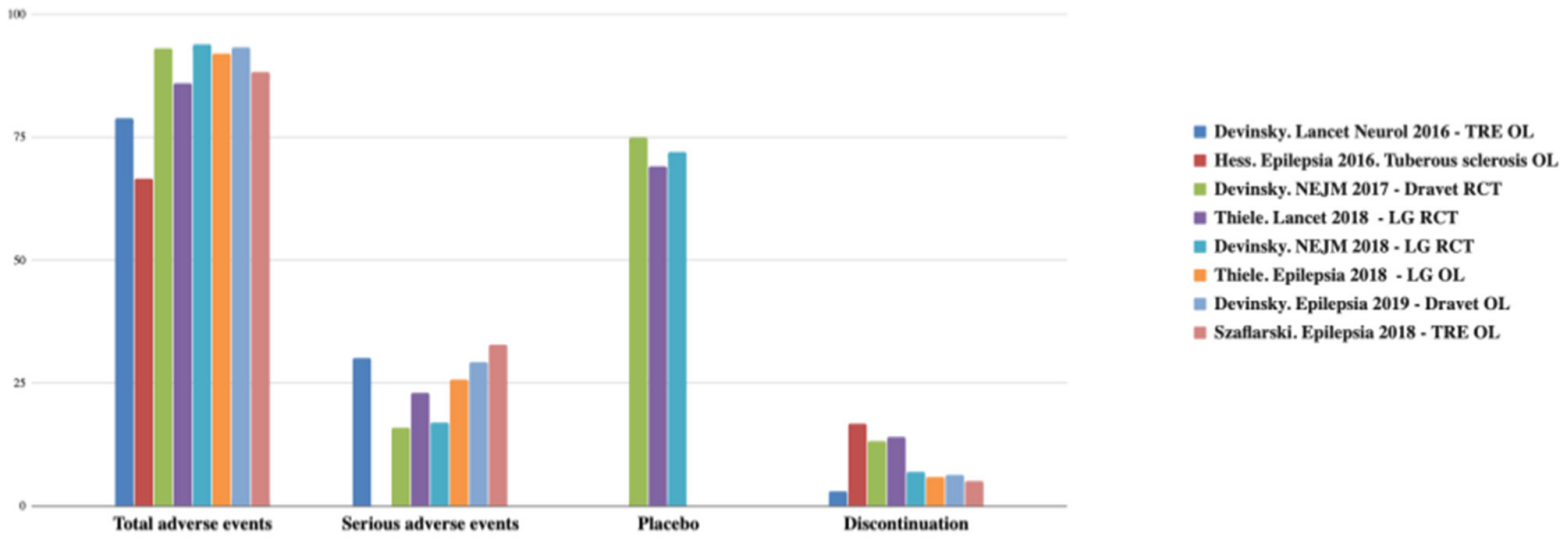
Total and serious adverse effects and discontinuation rates observed with CDB. TRE, treatment-resistant epilepsy; OL, open label; LG, Lennox-Gastaut; RCT, randomized controlled trial. Adapted from (12).

**Figure 3:**
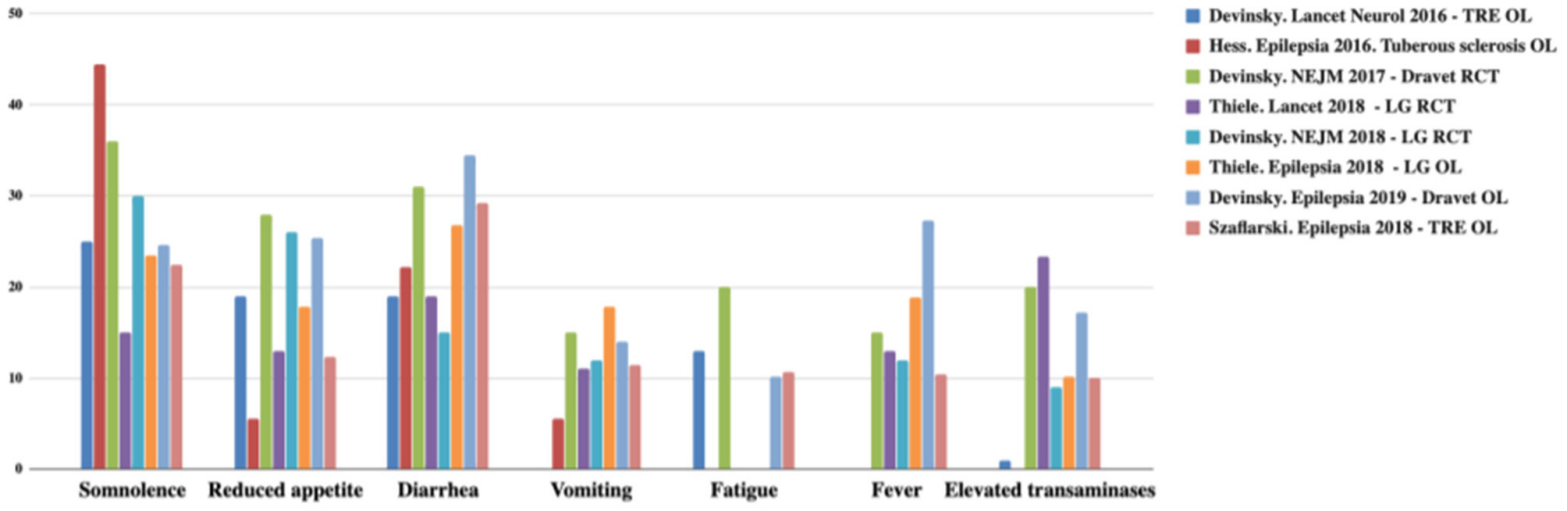
Most common adverse effects observed with CBD. TRE, treatment-resistant epilepsy; OL, open label; LG, Lennox-Gastaut; RCT, randomized controlled trial. Adapted from (12).

CBD has a better safety profile. Usually, adverse events (AEs) are mild and only observed in the first month. The most frequent AEs are drowsiness, reduced appetite, diarrhea, vomiting, fatigue, and fever. The CBD discontinuity rate is low (3–13%) (22–26, 34). AEs are summarized in Figures 2, 3 (12).

Laboratory monitoring is important for checking for hepatotoxicity. Increased transaminases lead to CBD withdrawal in a minority of patients. Concomitant use of valproate is a risk factor for this complication. Transaminases usually return to baseline after CBD dose reduction or withdrawal (24–26).

## Pharmacokinetics and Drug Interactions

As CBD has a half-life that ranges from 18 to 32 h, it is usually administered in two equally divided doses daily. CBD is highly lipophilic and is, therefore, sold in oil-based capsules or suspensions. Patients fed high-fat food presented a 14-fold higher serum concentration of CBD than those in a fasting condition (35). Thus, we recommend that CBD should be taken with food to maximize absorption.

CBD is included among the enzyme-inhibitor ASMs (36). Hence, clinically relevant drug interactions of CBD with other ASMs are a concern. CBD is a potent CYP3A4 and CYP2C19 inhibitor, which are responsible for the metabolism of CLB and N-desmethylclobazam (N-CLB), respectively (29). The combination CBD-CLB has been shown to increase CLB levels by an average of 60% (range ± 80%) after 4 weeks of initiation. An even higher increase occurs in N-desmethylclobazam (up to 500%), an active metabolite (37). This may increase both the effectiveness and the risk of AE for this combination of drugs.

CBD coadministration is also associated with an increase in zonisamide, eslicarbazepine, topiramate, and rufinamide levels (38). No interactions with valproic acid, stiripentol, and levetiracetam have been observed. Phenytoin and carbamazepine likely reduce CBD levels (39). Hence, drug therapeutic monitoring of other ASMs might be important when prescribing CBD as adjunctive therapy.

The relationship between CBD plasma level and seizure control is only partially known. There is a positive linear correlation between CBD dosage (range 5–50 mg/kg/day) and CBD plasma level (range 7.1–1,200 ng/mL). A 100 ng/mL increase in CBD level was shown to be associated with an approximately two count reduction in seizure frequency per time period (32).

## Gaps in Knowledge: CBD for Epilepsy in Adults, Long-Term Safety, and Access to CBD

Adult patients with epilepsy are underrepresented in most CBD trials. Common etiologies for focal epilepsy in adults, such as poststroke epilepsy, traumatic brain injury, and focal cortical dysplasia, are not properly represented in these trials.

Long-term cognitive, behavioral, and psychiatric side effects are another concern. Cannabis use is associated with executive dysfunction. This may impair worker and driver safety. Depression, psychosis, and suicide are more common among regular cannabis users (40).

As expected for a new ASM, there are no clinical data about the teratogenicity of CBD. However, prenatal cannabis is associated with low birth weight (41).

In children the recommended starting dosage is 2.5 mg/kg taken twice daily (5 mg/kg/day). After 1 week, the dosage can be increased to a maintenance dosage of 5 mg/kg twice daily (10 mg/kg/day). The dosage could be increased weekly in increments of 2.5 mg/kg twice daily (5 mg/kg/day) up to a maximum recommended dose of 10 mg/kg twice daily (20 mg/kg/day) based on clinical response and tolerability according to the label (42, 43). There is published data suggesting that dosage could be increased up to 50 mg/kg/day if tolerated and clinical response is observed (22, 23, 44).

However, for patients with the same CBD plasma level, differences in seizure improvement do not depend on age. Thus, plasma levels may be a strategy to guide CBD doses for adult patients with epilepsy. Assays have been developed and validated and may be of clinical utility in the future (32, 45, 46).

Tolerance may occur in up to one third of patients with CBD. It can be defined as either the necessity to increase the dose by at least 30% or a response reduction of more than 30%. The mean time until the appearance of tolerance was found to be 7 months. The long-term risks and efficacy of CBD use are unknown (47).

Legal aspects of cannabis cultivation, manufacture, and distribution limit CBD use. Cannabis-based products usually present low concentrations of CBD, and almost all contain THC (48, 49). Although an FDA-approved, pharmaceutical-grade, high-purity CBD is available (Epidiolex®) (42, 43), its cost is prohibitive for some patients. A synthetic CBD may be a solution to legal obstacles and costs (50).

## Conclusion

CBD is an effective treatment for patients with Dravet and Lennox-Gastaut syndromes. The use of cannabis and other THC-rich products is not appropriate because of its dubious effect on seizure control and negative psychotropic effects (12).

The role of CBD in the treatment of drug-resistant epilepsy is adjuvant and often overstated. Adverse effects are usually mild, and the discontinuation rate is low. Laboratory monitoring should focus on hepatotoxicity and therapeutic drug monitoring of concomitant ASMs, especially CLB.

Topics for future research should include (a) the efficacy and optimal dose of CBD for adult patients with epilepsy, especially focal epilepsy; (b) long-term psychiatric and cognitive adverse effects related to CBD; and (c) strategies to reduce costs and improve access to CBD for people living with epilepsy (50).

## Author's Note

This is an adapted English language translation of ‘Análise crítica do papel do canabidiol no tratamento da epilepsia,' originally published in ‘Epilepsia Atual.' LP prepared this translation with support from other authors. Permission was granted by Editora OMNIFARMA.

## Author Contributions

GS and FD: drafting, editing and organizing of the manuscript. MO: literature review and drafting of the manuscript. LP: drafting and revision of the manuscript. All authors contributed to the article and approved the submitted version.

## Conflict of Interest

LP received speaker or consultancy fees from UCB, Livanova, Genom – União Química, Zodiac and United Medical. The remaining authors declare that the research was conducted in the absence of any commercial or financial relationships that could be construed as a potential conflict of interest.
